# 

**Published:** 1913-06

**Authors:** 


### CONTENTS.

ORIGINAL COMMUNICATIONS—

The Proper Care of Milk in the Home. By Claude A. Smith, M. D., Atlanta, Ga. ............... 97

The Value of and Indications for Incision of Ear Drum in Otitis Media. By Richard M. Nelson, M. D., Atlanta, Ga. ................... 106

EDITORIAL ................................. 115

SELECTIONS AND ABSTRACTS ................. 119

BOOK REVIEWS ............................. 142

